# 2,13-Dibenzyl-5,16-diethyl-2,6,13,17-tetra­aza­tricyclo­[16.4.0.0^7,12^]docosan-2-ium nitrate

**DOI:** 10.1107/S1600536811029692

**Published:** 2011-07-30

**Authors:** Jong-Ha Choi, Md Abdus Subhan, Seik Weng Ng, Edward R. T. Tiekink

**Affiliations:** a Department of Chemistry, Andong National University, Andong 760-749, Republic of Korea; b Department of Chemistry, University of Malaya, 50603 Kuala Lumpur, Malaysia; c Chemistry Department, Faculty of Science, King Abdulaziz University, PO Box 80203 Jeddah, Saudi Arabia

## Abstract

One of the tertiary amine atoms has been protonated in the title salt, C_36_H_57_N_4_^+^·NO_3_^−^. The four N atoms of the macrocycle are almost coplanar (r.m.s. deviation = 0.0053 Å), a result correlated with the formation of intra­molecular N—H⋯N and N—H⋯(N,N) hydrogen bonds. With respect to this plane, the benzyl groups lie to either side; a similar arrangement pertains for the cyclo­hexyl rings (each with a chair conformation). Helical supra­molecular chains are evident in the crystal, whereby alternating cations and anions are linked by C—H⋯O inter­actions. The chains are consolidated into supra­molecular arrays in the *ab* plane *via* C—H⋯π contacts involving both benzene rings.

## Related literature

For the synthesis of the precursor macrocycle, see: Lim *et al.* (2006 ▶); For related structures, see: Choi *et al.* (2006 ▶, 2010*a* ▶,*b* ▶).
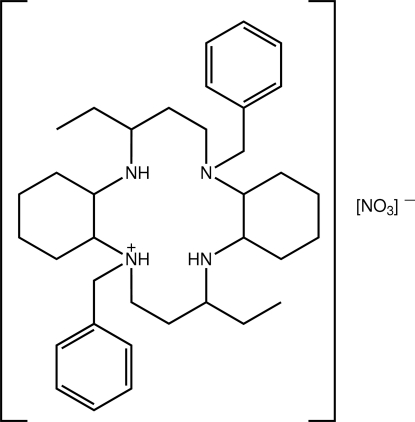


## Experimental

### 

#### Crystal data

C_36_H_57_N_4_^+^·NO_3_^−^
*M* *_r_* = 607.87
Monoclinic, 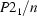

*a* = 10.7882 (1) Å
*b* = 16.2785 (1) Å
*c* = 19.0962 (1) Å
β = 98.2461 (6)°
*V* = 3318.92 (4) Å^3^
*Z* = 4
Cu *K*α radiation
μ = 0.61 mm^−1^
*T* = 100 K
0.30 × 0.20 × 0.10 mm

#### Data collection

Agilent SuperNova Dual diffractometer with an Atlas detector
Absorption correction: multi-scan (*CrysAlis PRO*; Agilent, 2010 ▶) *T*_min_ = 0.839, *T*_max_ = 0.942
24730 measured reflections
6689 independent reflections
6289 reflections with *I* > 2σ(*I*)
*R*_int_ = 0.040

#### Refinement

*R*[*F* ^2^ > 2σ(*F* ^2^)] = 0.039
*wR*(*F* ^2^) = 0.120
*S* = 1.06
6689 reflections
409 parameters
3 restraints
H atoms treated by a mixture of independent and constrained refinement
Δρ_max_ = 0.31 e Å^−3^
Δρ_min_ = −0.25 e Å^−3^

### 

Data collection: *CrysAlis PRO* (Agilent, 2010 ▶); cell refinement: *CrysAlis PRO*; data reduction: *CrysAlis PRO*; program(s) used to solve structure: *SHELXS97* (Sheldrick, 2008 ▶); program(s) used to refine structure: *SHELXL97* (Sheldrick, 2008 ▶); molecular graphics: *ORTEP-3* (Farrugia, 1997 ▶) and *DIAMOND* (Brandenburg, 2006 ▶); software used to prepare material for publication: *publCIF* (Westrip, 2010 ▶).

## Supplementary Material

Crystal structure: contains datablock(s) global, I. DOI: 10.1107/S1600536811029692/hb6328sup1.cif

Structure factors: contains datablock(s) I. DOI: 10.1107/S1600536811029692/hb6328Isup2.hkl

Supplementary material file. DOI: 10.1107/S1600536811029692/hb6328Isup3.cml

Additional supplementary materials:  crystallographic information; 3D view; checkCIF report

## Figures and Tables

**Table 1:** Hydrogen-bond geometry (Å, °) *Cg*1 and *Cg*2 are the centroids of the C2–C7 and C20–C25 benzene rings, respectively.

*D*—H⋯*A*	*D*—H	H⋯*A*	*D*⋯*A*	*D*—H⋯*A*
N1—H1⋯N2	0.90 (1)	2.32 (1)	2.7400 (11)	108 (1)
N1—H1⋯N4	0.90 (1)	2.12 (1)	2.8156 (11)	134 (1)
N2—H2⋯N3	0.88 (1)	2.19 (1)	2.9293 (11)	142 (1)
N4—H4⋯N3	0.88 (1)	2.33 (1)	2.7992 (11)	113 (1)
C1—H1a⋯O1	0.99	2.36	3.2096 (13)	143
C9—H9a⋯O3^i^	0.99	2.40	3.3620 (12)	165
C34—H34a⋯O3^i^	0.99	2.50	3.3876 (13)	150
C8—H8a⋯*Cg*3^ii^	0.99	2.53	3.4008 (11)	146
C26—H26b⋯*Cg*1^iii^	0.99	2.71	3.5899 (11)	149

Symmetry codes: (i) 
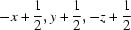
; (ii) 
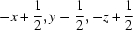
; (iii) 
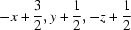
.

**Fig. 1.**
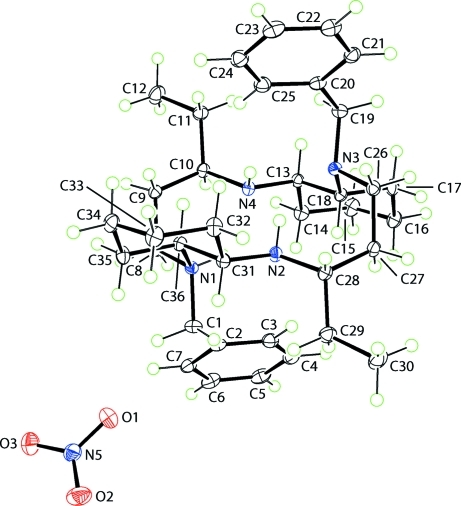
Molecular structures of the ions in (I) showing displacement ellipsoids at the 50% probability level.

**Fig. 2.**
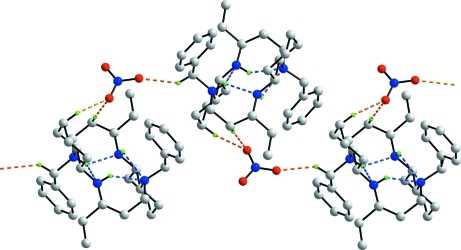
Helical supramolecular chain aligned along the b-axis in (I) mediated by C—H···O interactions shown as orange dashed lines. Intramolecular N—H···N hydrogen bonds are shown as blue dashed lines.

**Fig. 3.**
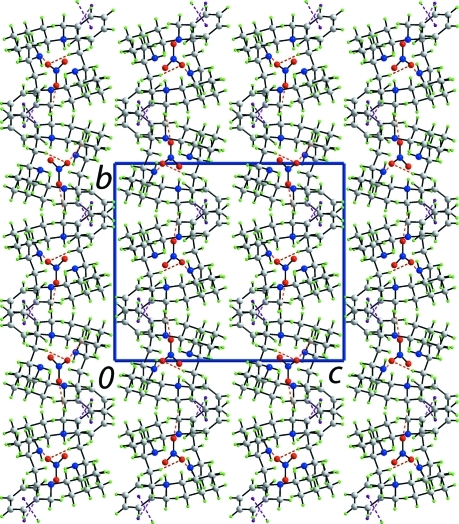
A view of the crystal packing of (I) in projection down the a-axis. The C—H···O and C—H···π interactions shown as orange and purple dashed lines, respectively.

## supplementary crystallographic information

### Comment

The title salt, (I), was isolated unexpectedly during the course of studies of partially *N*-substituted tetraazamacrocycles of interest owing to their various applications (Choi *et al.*, 2006; Choi *et al.*, 2010*a*; Choi, *et al.*, 2010*b*). As seen in Fig. 1, one of the tertiary amine-N atoms, *i.e*. N1, has been protonated with the charge balance provided by the nitrate anion. The four nitrogen atoms lie in a plane with a r.m.s. deviation = 0.0053 Å; the maximum deviation from the least-squares plane is 0.0055 (4) Å for atom N1. This observation is readily explained in terms of the intramolecular N—H···N hydrogen bonds with the N1—H1 atom being bifurcated, Table 1 and Fig. 2. With reference to this plane, the benzyl groups lie to either side and are twisted with respect to the N_4_ plane as seen in the values of the dihedral angles of 63.62 (3) and 66.25 (3) ° formed with rings (C2—C7) and (C20—C25), respectively. Similarly, the cyclohexyl rings, each with a chair conformation, lie to either side of the N_4_ plane.

The anion is associated with the cation *via* C—H···O contacts, Table 1, so that the nitrate-O1 forms a contact with a benzyl-methylene-H, and the nitrate-O3 atom bridges a methylene-H derived from a cyclohexyl ring and a methylene-H from the macrocyclic framework. The result is the formation of a helical supramolecular chain along the *b*-axis, Fig. 2. Chains are consolidated into layers in the *ab*-plane *via* C—H···π interactions involving benzene rings, Table 1 and Fig. 3.

### Experimental

The macrocycle, 5,16-diethyl-2,6,13,17-tetraazatricyclo[14.4.0^1,18^.0^7,12^]docosane, was prepared according to a published procedure (Lim *et al.*, 2006). To a solution of this macrocycle (0.61 g, 2.0 mmol) in methanol (10 ml) was added benzyl bromide (0.68 g, 4.0 mmol) and a solution containing sodium carbonate (0.42 g, 4.0 mmol) in water (5 ml). The mixture was refluxed for 24 h. The solution was cooled, the white solid collected and washed with water. The title di-benzyl substituted macrocyclic nitrate was the unexpected colourless by-product that was obtained when copper nitrate trihydrate (0.06 g, 0.25 mmol) and the dibenzyl-substituted macrocycle (0.16 g, 0.29 mmol) was reacted in THF (10 ml). The compound was recrystallized from acetonitrile-water (1:1) in the form of colourless prisms.

### Refinement

Carbon-bound H-atoms were placed in calculated positions [C—H 0.95 to 0.98 Å, *U*_iso_(H) 1.2 to 1.5*U*_eq_(C)] and were included in the refinement in the riding model approximation. The amino H-atoms were located in a difference Fourier map, and were refined with a distance restraint of N–H 0.88±0.01 Å; their *U*_iso_ values were refined.

### Crystal data

**Table:**

C_36_H_57_N_4_^+^·NO_3_^−^	*F*(000) = 1328
*M**_r_* = 607.87	*D*_x_ = 1.217 Mg m^−^^3^
Monoclinic, *P*2_1_/*n*	Cu *K*α radiation, λ = 1.54184 Å
Hall symbol: -P 2yn	Cell parameters from 15422 reflections
*a* = 10.7882 (1) Å	θ = 2.7–74.2°
*b* = 16.2785 (1) Å	µ = 0.61 mm^−^^1^
*c* = 19.0962 (1) Å	*T* = 100 K
β = 98.2461 (6)°	Prism, colorless
*V* = 3318.92 (4) Å^3^	0.30 × 0.20 × 0.10 mm
*Z* = 4	

### Data collection

**Table:**

Agilent SuperNova Dual diffractometer with an Atlas detector	6689 independent reflections
Radiation source: SuperNova (Cu) X-ray Source	6289 reflections with *I* > 2σ(*I*)
Mirror	*R*_int_ = 0.040
Detector resolution: 10.4041 pixels mm^-1^	θ_max_ = 74.4°, θ_min_ = 3.6°
ω scans	*h* = −13→13
Absorption correction: multi-scan (*CrysAlis PRO*; Agilent, 2010)	*k* = −20→20
*T*_min_ = 0.839, *T*_max_ = 0.942	*l* = −23→23
24730 measured reflections	

### Refinement

**Table:**

Refinement on *F*^2^	Primary atom site location: structure-invariant direct methods
Least-squares matrix: full	Secondary atom site location: difference Fourier map
*R*[*F*^2^ > 2σ(*F*^2^)] = 0.039	Hydrogen site location: inferred from neighbouring sites
*wR*(*F*^2^) = 0.120	H atoms treated by a mixture of independent and constrained refinement
*S* = 1.06	*w* = 1/[σ^2^(*F*_o_^2^) + (0.0639*P*)^2^ + 0.9655*P*] where *P* = (*F*_o_^2^ + 2*F*_c_^2^)/3
6689 reflections	(Δ/σ)_max_ = 0.001
409 parameters	Δρ_max_ = 0.31 e Å^−^^3^
3 restraints	Δρ_min_ = −0.25 e Å^−^^3^

### Special details

**Table:**

Geometry. All e.s.d.'s (except the e.s.d. in the dihedral angle between two l.s. planes) are estimated using the full covariance matrix. The cell e.s.d.'s are taken into account individually in the estimation of e.s.d.'s in distances, angles and torsion angles; correlations between e.s.d.'s in cell parameters are only used when they are defined by crystal symmetry. An approximate (isotropic) treatment of cell e.s.d.'s is used for estimating e.s.d.'s involving l.s. planes.

### Fractional atomic coordinates and isotropic or equivalent isotropic displacement parameters (Å^2^)

**Table:**

	*x*	*y*	*z*	*U*_iso_*/*U*_eq_	
O1	0.50228 (8)	0.10547 (5)	0.24392 (5)	0.0332 (2)	
O2	0.56328 (8)	−0.01583 (6)	0.28070 (5)	0.0327 (2)	
O3	0.39115 (8)	−0.00135 (5)	0.20754 (4)	0.02779 (19)	
N1	0.43505 (7)	0.37105 (5)	0.22433 (4)	0.01185 (17)	
N2	0.57885 (7)	0.45766 (5)	0.32911 (4)	0.01289 (18)	
N3	0.56192 (7)	0.62777 (5)	0.27861 (4)	0.01193 (17)	
N4	0.41868 (7)	0.53381 (5)	0.17396 (4)	0.01209 (17)	
N5	0.48585 (8)	0.02911 (6)	0.24405 (5)	0.0209 (2)	
C1	0.52521 (9)	0.30042 (6)	0.22417 (5)	0.0158 (2)	
H1A	0.4785	0.2479	0.2221	0.019*	
H1B	0.5853	0.3011	0.2685	0.019*	
C2	0.59572 (9)	0.30616 (6)	0.16165 (5)	0.0148 (2)	
C3	0.69049 (9)	0.36427 (6)	0.16127 (5)	0.0176 (2)	
H3A	0.7107	0.4000	0.2006	0.021*	
C4	0.75561 (10)	0.37032 (7)	0.10379 (6)	0.0207 (2)	
H4A	0.8200	0.4101	0.1037	0.025*	
C5	0.72593 (10)	0.31770 (7)	0.04624 (5)	0.0212 (2)	
H5	0.7700	0.3218	0.0067	0.025*	
C6	0.63240 (10)	0.25951 (7)	0.04648 (5)	0.0205 (2)	
H6	0.6127	0.2236	0.0072	0.025*	
C7	0.56721 (9)	0.25352 (7)	0.10399 (5)	0.0175 (2)	
H7	0.5031	0.2135	0.1040	0.021*	
C8	0.33318 (9)	0.36493 (6)	0.16142 (5)	0.0138 (2)	
H8A	0.2848	0.3140	0.1659	0.017*	
H8B	0.3729	0.3601	0.1180	0.017*	
C9	0.24291 (9)	0.43716 (6)	0.15289 (5)	0.0135 (2)	
H9A	0.2132	0.4475	0.1988	0.016*	
H9B	0.1691	0.4220	0.1183	0.016*	
C10	0.29874 (9)	0.51689 (6)	0.12824 (5)	0.0129 (2)	
H10	0.3172	0.5080	0.0790	0.015*	
C11	0.20492 (9)	0.58771 (6)	0.12639 (5)	0.0171 (2)	
H11A	0.1853	0.5968	0.1749	0.020*	
H11B	0.2448	0.6383	0.1116	0.020*	
C12	0.08247 (10)	0.57368 (8)	0.07684 (6)	0.0254 (3)	
H12A	0.0277	0.6215	0.0785	0.038*	
H12B	0.0407	0.5246	0.0919	0.038*	
H12C	0.1004	0.5660	0.0284	0.038*	
C13	0.49956 (9)	0.59796 (6)	0.14997 (5)	0.0125 (2)	
H13	0.4500	0.6496	0.1400	0.015*	
C14	0.55226 (9)	0.57130 (6)	0.08313 (5)	0.0162 (2)	
H14A	0.4824	0.5636	0.0440	0.019*	
H14B	0.5959	0.5180	0.0919	0.019*	
C15	0.64389 (10)	0.63526 (7)	0.06136 (5)	0.0192 (2)	
H15A	0.6804	0.6146	0.0201	0.023*	
H15B	0.5979	0.6866	0.0471	0.023*	
C16	0.74889 (9)	0.65372 (7)	0.12163 (5)	0.0184 (2)	
H16A	0.8027	0.6979	0.1070	0.022*	
H16B	0.8013	0.6041	0.1322	0.022*	
C17	0.69583 (9)	0.68032 (6)	0.18819 (5)	0.0163 (2)	
H17A	0.6492	0.7325	0.1789	0.020*	
H17B	0.7653	0.6898	0.2272	0.020*	
C18	0.60803 (9)	0.61374 (6)	0.21000 (5)	0.0120 (2)	
H18	0.6581	0.5619	0.2156	0.014*	
C19	0.48660 (9)	0.70312 (6)	0.27985 (5)	0.0147 (2)	
H19A	0.4286	0.7079	0.2349	0.018*	
H19B	0.5426	0.7516	0.2841	0.018*	
C20	0.41211 (9)	0.70173 (6)	0.34134 (5)	0.0145 (2)	
C21	0.44026 (9)	0.75553 (6)	0.39824 (5)	0.0171 (2)	
H21	0.5065	0.7940	0.3984	0.020*	
C22	0.37204 (10)	0.75333 (7)	0.45488 (5)	0.0196 (2)	
H22	0.3917	0.7904	0.4933	0.023*	
C23	0.27563 (10)	0.69726 (7)	0.45545 (5)	0.0196 (2)	
H23	0.2296	0.6956	0.4943	0.024*	
C24	0.24625 (10)	0.64323 (7)	0.39889 (6)	0.0197 (2)	
H24	0.1801	0.6047	0.3990	0.024*	
C25	0.31418 (9)	0.64595 (7)	0.34239 (5)	0.0172 (2)	
H25	0.2936	0.6092	0.3038	0.021*	
C26	0.66397 (9)	0.63019 (6)	0.33960 (5)	0.0141 (2)	
H26A	0.6256	0.6364	0.3834	0.017*	
H26B	0.7152	0.6798	0.3350	0.017*	
C27	0.75144 (9)	0.55589 (6)	0.34862 (5)	0.0141 (2)	
H27A	0.8269	0.5709	0.3820	0.017*	
H27B	0.7789	0.5439	0.3024	0.017*	
C28	0.69645 (9)	0.47719 (6)	0.37546 (5)	0.0137 (2)	
H28	0.6780	0.4864	0.4247	0.016*	
C29	0.78903 (9)	0.40598 (7)	0.37584 (5)	0.0182 (2)	
H29A	0.7483	0.3551	0.3895	0.022*	
H29B	0.8088	0.3982	0.3272	0.022*	
C30	0.91137 (10)	0.41840 (8)	0.42597 (6)	0.0260 (3)	
H30A	0.9657	0.3706	0.4235	0.039*	
H30B	0.8931	0.4248	0.4745	0.039*	
H30C	0.9537	0.4678	0.4121	0.039*	
C31	0.49519 (9)	0.39720 (6)	0.35440 (5)	0.0128 (2)	
H31	0.5424	0.3446	0.3638	0.015*	
C32	0.43876 (9)	0.42032 (7)	0.42130 (5)	0.0171 (2)	
H32A	0.5070	0.4289	0.4611	0.021*	
H32B	0.3916	0.4724	0.4131	0.021*	
C33	0.35099 (10)	0.35262 (7)	0.44074 (5)	0.0196 (2)	
H33A	0.4000	0.3021	0.4536	0.023*	
H33B	0.3128	0.3701	0.4825	0.023*	
C34	0.24755 (9)	0.33400 (7)	0.37957 (5)	0.0179 (2)	
H34A	0.1921	0.3824	0.3707	0.021*	
H34B	0.1965	0.2874	0.3927	0.021*	
C35	0.30172 (9)	0.31249 (6)	0.31183 (5)	0.0169 (2)	
H35A	0.3502	0.2608	0.3187	0.020*	
H35B	0.2329	0.3045	0.2722	0.020*	
C36	0.38655 (9)	0.38224 (6)	0.29457 (5)	0.0123 (2)	
H36	0.3345	0.4333	0.2903	0.015*	
H1	0.4755 (12)	0.4183 (6)	0.2191 (7)	0.030 (4)*	
H2	0.5374 (12)	0.5033 (6)	0.3183 (7)	0.029 (4)*	
H4	0.4029 (12)	0.5486 (8)	0.2162 (5)	0.023 (3)*	

### Atomic displacement parameters (Å^2^)

**Table:**

	*U*^11^	*U*^22^	*U*^33^	*U*^12^	*U*^13^	*U*^23^
O1	0.0302 (4)	0.0194 (4)	0.0498 (5)	−0.0036 (3)	0.0049 (4)	0.0009 (4)
O2	0.0298 (5)	0.0338 (5)	0.0338 (5)	0.0102 (4)	0.0020 (4)	0.0097 (4)
O3	0.0268 (4)	0.0291 (4)	0.0266 (4)	−0.0065 (3)	0.0007 (3)	−0.0020 (3)
N1	0.0122 (4)	0.0118 (4)	0.0116 (4)	0.0002 (3)	0.0018 (3)	−0.0005 (3)
N2	0.0117 (4)	0.0136 (4)	0.0129 (4)	−0.0015 (3)	0.0001 (3)	0.0025 (3)
N3	0.0116 (4)	0.0130 (4)	0.0110 (4)	0.0007 (3)	0.0010 (3)	−0.0007 (3)
N4	0.0119 (4)	0.0143 (4)	0.0099 (4)	−0.0016 (3)	0.0010 (3)	0.0004 (3)
N5	0.0194 (4)	0.0223 (5)	0.0225 (4)	0.0007 (3)	0.0078 (3)	0.0010 (3)
C1	0.0175 (5)	0.0145 (5)	0.0156 (5)	0.0049 (4)	0.0037 (4)	0.0013 (4)
C2	0.0135 (4)	0.0169 (5)	0.0140 (4)	0.0048 (4)	0.0014 (3)	0.0009 (4)
C3	0.0174 (5)	0.0174 (5)	0.0176 (5)	0.0029 (4)	0.0013 (4)	−0.0021 (4)
C4	0.0170 (5)	0.0209 (5)	0.0249 (5)	0.0017 (4)	0.0054 (4)	0.0034 (4)
C5	0.0194 (5)	0.0294 (6)	0.0158 (5)	0.0079 (4)	0.0059 (4)	0.0038 (4)
C6	0.0179 (5)	0.0285 (6)	0.0142 (5)	0.0060 (4)	−0.0006 (4)	−0.0040 (4)
C7	0.0132 (4)	0.0206 (5)	0.0180 (5)	0.0031 (4)	0.0005 (4)	−0.0026 (4)
C8	0.0137 (4)	0.0142 (5)	0.0127 (4)	−0.0004 (4)	−0.0004 (3)	−0.0015 (3)
C9	0.0118 (4)	0.0142 (5)	0.0142 (4)	−0.0002 (3)	0.0003 (3)	0.0001 (3)
C10	0.0122 (4)	0.0149 (5)	0.0109 (4)	−0.0008 (4)	−0.0003 (3)	0.0004 (3)
C11	0.0149 (5)	0.0163 (5)	0.0193 (5)	0.0017 (4)	0.0005 (4)	0.0029 (4)
C12	0.0156 (5)	0.0270 (6)	0.0316 (6)	0.0006 (4)	−0.0037 (4)	0.0086 (5)
C13	0.0125 (4)	0.0125 (5)	0.0124 (4)	−0.0008 (3)	0.0017 (3)	0.0013 (3)
C14	0.0171 (5)	0.0196 (5)	0.0122 (4)	−0.0008 (4)	0.0034 (4)	−0.0003 (4)
C15	0.0193 (5)	0.0249 (6)	0.0145 (5)	−0.0019 (4)	0.0059 (4)	0.0031 (4)
C16	0.0154 (5)	0.0223 (5)	0.0186 (5)	−0.0024 (4)	0.0064 (4)	0.0015 (4)
C17	0.0149 (5)	0.0173 (5)	0.0172 (5)	−0.0039 (4)	0.0044 (4)	−0.0002 (4)
C18	0.0113 (4)	0.0133 (5)	0.0116 (4)	−0.0002 (3)	0.0024 (3)	0.0002 (3)
C19	0.0156 (4)	0.0136 (5)	0.0150 (5)	0.0020 (4)	0.0030 (4)	0.0006 (3)
C20	0.0138 (4)	0.0158 (5)	0.0138 (4)	0.0041 (4)	0.0012 (3)	0.0006 (4)
C21	0.0133 (4)	0.0195 (5)	0.0178 (5)	0.0018 (4)	0.0001 (4)	−0.0022 (4)
C22	0.0189 (5)	0.0253 (6)	0.0134 (5)	0.0056 (4)	−0.0014 (4)	−0.0043 (4)
C23	0.0194 (5)	0.0258 (6)	0.0145 (5)	0.0067 (4)	0.0052 (4)	0.0035 (4)
C24	0.0181 (5)	0.0194 (5)	0.0223 (5)	0.0015 (4)	0.0057 (4)	0.0021 (4)
C25	0.0171 (5)	0.0176 (5)	0.0172 (5)	0.0014 (4)	0.0028 (4)	−0.0020 (4)
C26	0.0134 (4)	0.0147 (5)	0.0135 (4)	−0.0012 (4)	−0.0004 (4)	−0.0011 (3)
C27	0.0115 (4)	0.0159 (5)	0.0145 (4)	−0.0012 (4)	−0.0001 (3)	0.0009 (4)
C28	0.0125 (4)	0.0167 (5)	0.0111 (4)	−0.0004 (4)	−0.0004 (3)	0.0006 (3)
C29	0.0163 (5)	0.0186 (5)	0.0191 (5)	0.0024 (4)	0.0004 (4)	0.0033 (4)
C30	0.0157 (5)	0.0284 (6)	0.0322 (6)	0.0014 (4)	−0.0028 (4)	0.0095 (5)
C31	0.0130 (4)	0.0134 (5)	0.0120 (4)	−0.0008 (3)	0.0018 (3)	0.0009 (3)
C32	0.0187 (5)	0.0204 (5)	0.0125 (4)	−0.0027 (4)	0.0036 (4)	−0.0008 (4)
C33	0.0206 (5)	0.0249 (5)	0.0141 (5)	−0.0022 (4)	0.0056 (4)	0.0031 (4)
C34	0.0165 (5)	0.0203 (5)	0.0182 (5)	−0.0025 (4)	0.0069 (4)	0.0022 (4)
C35	0.0171 (5)	0.0173 (5)	0.0168 (5)	−0.0052 (4)	0.0046 (4)	−0.0011 (4)
C36	0.0124 (4)	0.0138 (5)	0.0112 (4)	−0.0004 (3)	0.0030 (3)	0.0001 (3)

### Geometric parameters (Å, °)

**Table:**

O1—N5	1.2557 (13)	C15—H15B	0.9900
O2—N5	1.2470 (13)	C16—C17	1.5297 (13)
O3—N5	1.2536 (12)	C16—H16A	0.9900
N1—C1	1.5063 (12)	C16—H16B	0.9900
N1—C8	1.5107 (12)	C17—C18	1.5357 (13)
N1—C36	1.5189 (11)	C17—H17A	0.9900
N1—H1	0.896 (9)	C17—H17B	0.9900
N2—C31	1.4634 (12)	C18—H18	1.0000
N2—C28	1.4737 (12)	C19—C20	1.5152 (13)
N2—H2	0.877 (9)	C19—H19A	0.9900
N3—C19	1.4734 (12)	C19—H19B	0.9900
N3—C26	1.4840 (12)	C20—C21	1.3947 (14)
N3—C18	1.4842 (11)	C20—C25	1.3954 (14)
N4—C13	1.4753 (12)	C21—C22	1.3933 (14)
N4—C10	1.4793 (11)	C21—H21	0.9500
N4—H4	0.881 (8)	C22—C23	1.3850 (16)
C1—C2	1.5077 (13)	C22—H22	0.9500
C1—H1A	0.9900	C23—C24	1.3937 (15)
C1—H1B	0.9900	C23—H23	0.9500
C2—C3	1.3935 (14)	C24—C25	1.3892 (14)
C2—C7	1.3943 (14)	C24—H24	0.9500
C3—C4	1.3890 (14)	C25—H25	0.9500
C3—H3A	0.9500	C26—C27	1.5286 (13)
C4—C5	1.3940 (16)	C26—H26A	0.9900
C4—H4A	0.9500	C26—H26B	0.9900
C5—C6	1.3844 (16)	C27—C28	1.5308 (13)
C5—H5	0.9500	C27—H27A	0.9900
C6—C7	1.3898 (14)	C27—H27B	0.9900
C6—H6	0.9500	C28—C29	1.5295 (14)
C7—H7	0.9500	C28—H28	1.0000
C8—C9	1.5204 (13)	C29—C30	1.5287 (14)
C8—H8A	0.9900	C29—H29A	0.9900
C8—H8B	0.9900	C29—H29B	0.9900
C9—C10	1.5331 (13)	C30—H30A	0.9800
C9—H9A	0.9900	C30—H30B	0.9800
C9—H9B	0.9900	C30—H30C	0.9800
C10—C11	1.5312 (13)	C31—C36	1.5344 (12)
C10—H10	1.0000	C31—C32	1.5387 (13)
C11—C12	1.5277 (14)	C31—H31	1.0000
C11—H11A	0.9900	C32—C33	1.5328 (14)
C11—H11B	0.9900	C32—H32A	0.9900
C12—H12A	0.9800	C32—H32B	0.9900
C12—H12B	0.9800	C33—C34	1.5256 (14)
C12—H12C	0.9800	C33—H33A	0.9900
C13—C14	1.5326 (13)	C33—H33B	0.9900
C13—C18	1.5378 (12)	C34—C35	1.5344 (13)
C13—H13	1.0000	C34—H34A	0.9900
C14—C15	1.5336 (14)	C34—H34B	0.9900
C14—H14A	0.9900	C35—C36	1.5238 (13)
C14—H14B	0.9900	C35—H35A	0.9900
C15—C16	1.5245 (14)	C35—H35B	0.9900
C15—H15A	0.9900	C36—H36	1.0000
			
C1—N1—C8	110.14 (7)	C16—C17—H17B	109.6
C1—N1—C36	113.44 (7)	C18—C17—H17B	109.6
C8—N1—C36	114.00 (7)	H17A—C17—H17B	108.1
C1—N1—H1	109.2 (9)	N3—C18—C17	115.38 (8)
C8—N1—H1	106.1 (9)	N3—C18—C13	111.61 (7)
C36—N1—H1	103.4 (9)	C17—C18—C13	110.41 (8)
C31—N2—C28	117.74 (7)	N3—C18—H18	106.3
C31—N2—H2	109.3 (9)	C17—C18—H18	106.3
C28—N2—H2	108.9 (9)	C13—C18—H18	106.3
C19—N3—C26	108.30 (7)	N3—C19—C20	110.80 (8)
C19—N3—C18	113.43 (7)	N3—C19—H19A	109.5
C26—N3—C18	113.09 (7)	C20—C19—H19A	109.5
C13—N4—C10	117.05 (7)	N3—C19—H19B	109.5
C13—N4—H4	106.9 (9)	C20—C19—H19B	109.5
C10—N4—H4	108.9 (9)	H19A—C19—H19B	108.1
O2—N5—O3	120.46 (10)	C21—C20—C25	118.57 (9)
O2—N5—O1	119.90 (9)	C21—C20—C19	120.93 (9)
O3—N5—O1	119.64 (9)	C25—C20—C19	120.51 (9)
N1—C1—C2	110.71 (8)	C22—C21—C20	120.56 (10)
N1—C1—H1A	109.5	C22—C21—H21	119.7
C2—C1—H1A	109.5	C20—C21—H21	119.7
N1—C1—H1B	109.5	C23—C22—C21	120.26 (9)
C2—C1—H1B	109.5	C23—C22—H22	119.9
H1A—C1—H1B	108.1	C21—C22—H22	119.9
C3—C2—C7	119.47 (9)	C22—C23—C24	119.83 (9)
C3—C2—C1	120.00 (9)	C22—C23—H23	120.1
C7—C2—C1	120.53 (9)	C24—C23—H23	120.1
C4—C3—C2	120.45 (9)	C25—C24—C23	119.68 (10)
C4—C3—H3A	119.8	C25—C24—H24	120.2
C2—C3—H3A	119.8	C23—C24—H24	120.2
C3—C4—C5	119.63 (10)	C24—C25—C20	121.11 (9)
C3—C4—H4A	120.2	C24—C25—H25	119.4
C5—C4—H4A	120.2	C20—C25—H25	119.4
C6—C5—C4	120.20 (9)	N3—C26—C27	116.35 (8)
C6—C5—H5	119.9	N3—C26—H26A	108.2
C4—C5—H5	119.9	C27—C26—H26A	108.2
C5—C6—C7	120.15 (10)	N3—C26—H26B	108.2
C5—C6—H6	119.9	C27—C26—H26B	108.2
C7—C6—H6	119.9	H26A—C26—H26B	107.4
C6—C7—C2	120.10 (10)	C26—C27—C28	115.93 (8)
C6—C7—H7	120.0	C26—C27—H27A	108.3
C2—C7—H7	120.0	C28—C27—H27A	108.3
N1—C8—C9	114.58 (8)	C26—C27—H27B	108.3
N1—C8—H8A	108.6	C28—C27—H27B	108.3
C9—C8—H8A	108.6	H27A—C27—H27B	107.4
N1—C8—H8B	108.6	N2—C28—C29	110.17 (8)
C9—C8—H8B	108.6	N2—C28—C27	108.73 (7)
H8A—C8—H8B	107.6	C29—C28—C27	110.59 (8)
C8—C9—C10	114.44 (8)	N2—C28—H28	109.1
C8—C9—H9A	108.7	C29—C28—H28	109.1
C10—C9—H9A	108.7	C27—C28—H28	109.1
C8—C9—H9B	108.7	C30—C29—C28	114.03 (9)
C10—C9—H9B	108.7	C30—C29—H29A	108.7
H9A—C9—H9B	107.6	C28—C29—H29A	108.7
N4—C10—C11	113.13 (8)	C30—C29—H29B	108.7
N4—C10—C9	108.99 (7)	C28—C29—H29B	108.7
C11—C10—C9	110.91 (8)	H29A—C29—H29B	107.6
N4—C10—H10	107.9	C29—C30—H30A	109.5
C11—C10—H10	107.9	C29—C30—H30B	109.5
C9—C10—H10	107.9	H30A—C30—H30B	109.5
C12—C11—C10	114.31 (9)	C29—C30—H30C	109.5
C12—C11—H11A	108.7	H30A—C30—H30C	109.5
C10—C11—H11A	108.7	H30B—C30—H30C	109.5
C12—C11—H11B	108.7	N2—C31—C36	107.60 (7)
C10—C11—H11B	108.7	N2—C31—C32	116.47 (8)
H11A—C11—H11B	107.6	C36—C31—C32	107.83 (8)
C11—C12—H12A	109.5	N2—C31—H31	108.2
C11—C12—H12B	109.5	C36—C31—H31	108.2
H12A—C12—H12B	109.5	C32—C31—H31	108.2
C11—C12—H12C	109.5	C33—C32—C31	110.91 (8)
H12A—C12—H12C	109.5	C33—C32—H32A	109.5
H12B—C12—H12C	109.5	C31—C32—H32A	109.5
N4—C13—C14	111.39 (8)	C33—C32—H32B	109.5
N4—C13—C18	107.91 (7)	C31—C32—H32B	109.5
C14—C13—C18	109.56 (8)	H32A—C32—H32B	108.0
N4—C13—H13	109.3	C34—C33—C32	111.53 (8)
C14—C13—H13	109.3	C34—C33—H33A	109.3
C18—C13—H13	109.3	C32—C33—H33A	109.3
C13—C14—C15	111.35 (8)	C34—C33—H33B	109.3
C13—C14—H14A	109.4	C32—C33—H33B	109.3
C15—C14—H14A	109.4	H33A—C33—H33B	108.0
C13—C14—H14B	109.4	C33—C34—C35	111.45 (8)
C15—C14—H14B	109.4	C33—C34—H34A	109.3
H14A—C14—H14B	108.0	C35—C34—H34A	109.3
C16—C15—C14	111.55 (8)	C33—C34—H34B	109.3
C16—C15—H15A	109.3	C35—C34—H34B	109.3
C14—C15—H15A	109.3	H34A—C34—H34B	108.0
C16—C15—H15B	109.3	C36—C35—C34	109.00 (8)
C14—C15—H15B	109.3	C36—C35—H35A	109.9
H15A—C15—H15B	108.0	C34—C35—H35A	109.9
C15—C16—C17	110.92 (8)	C36—C35—H35B	109.9
C15—C16—H16A	109.5	C34—C35—H35B	109.9
C17—C16—H16A	109.5	H35A—C35—H35B	108.3
C15—C16—H16B	109.5	N1—C36—C35	113.28 (8)
C17—C16—H16B	109.5	N1—C36—C31	110.70 (7)
H16A—C16—H16B	108.0	C35—C36—C31	112.06 (8)
C16—C17—C18	110.15 (8)	N1—C36—H36	106.8
C16—C17—H17A	109.6	C35—C36—H36	106.8
C18—C17—H17A	109.6	C31—C36—H36	106.8
			
C8—N1—C1—C2	66.13 (10)	C26—N3—C19—C20	−70.03 (9)
C36—N1—C1—C2	−164.73 (8)	C18—N3—C19—C20	163.57 (8)
N1—C1—C2—C3	74.22 (11)	N3—C19—C20—C21	111.49 (10)
N1—C1—C2—C7	−105.98 (10)	N3—C19—C20—C25	−68.03 (11)
C7—C2—C3—C4	0.50 (15)	C25—C20—C21—C22	0.13 (15)
C1—C2—C3—C4	−179.69 (9)	C19—C20—C21—C22	−179.40 (9)
C2—C3—C4—C5	−0.15 (15)	C20—C21—C22—C23	0.30 (15)
C3—C4—C5—C6	−0.24 (16)	C21—C22—C23—C24	−0.43 (15)
C4—C5—C6—C7	0.29 (16)	C22—C23—C24—C25	0.12 (15)
C5—C6—C7—C2	0.05 (15)	C23—C24—C25—C20	0.32 (15)
C3—C2—C7—C6	−0.45 (15)	C21—C20—C25—C24	−0.44 (15)
C1—C2—C7—C6	179.74 (9)	C19—C20—C25—C24	179.09 (9)
C1—N1—C8—C9	−175.10 (8)	C19—N3—C26—C27	178.66 (8)
C36—N1—C8—C9	56.07 (10)	C18—N3—C26—C27	−54.73 (11)
N1—C8—C9—C10	71.93 (10)	N3—C26—C27—C28	−73.19 (10)
C13—N4—C10—C11	−68.49 (10)	C31—N2—C28—C29	73.32 (10)
C13—N4—C10—C9	167.63 (8)	C31—N2—C28—C27	−165.34 (8)
C8—C9—C10—N4	−50.59 (10)	C26—C27—C28—N2	53.90 (10)
C8—C9—C10—C11	−175.77 (8)	C26—C27—C28—C29	174.98 (8)
N4—C10—C11—C12	177.23 (8)	N2—C28—C29—C30	−176.58 (8)
C9—C10—C11—C12	−59.95 (11)	C27—C28—C29—C30	63.19 (11)
C10—N4—C13—C14	−67.86 (10)	C28—N2—C31—C36	−175.09 (8)
C10—N4—C13—C18	171.85 (7)	C28—N2—C31—C32	63.80 (11)
N4—C13—C14—C15	−175.82 (8)	N2—C31—C32—C33	178.67 (8)
C18—C13—C14—C15	−56.50 (10)	C36—C31—C32—C33	57.68 (10)
C13—C14—C15—C16	55.23 (11)	C31—C32—C33—C34	−56.30 (11)
C14—C15—C16—C17	−55.12 (12)	C32—C33—C34—C35	54.94 (12)
C15—C16—C17—C18	56.98 (11)	C33—C34—C35—C36	−55.57 (11)
C19—N3—C18—C17	61.89 (10)	C1—N1—C36—C35	−67.88 (10)
C26—N3—C18—C17	−61.94 (10)	C8—N1—C36—C35	59.27 (10)
C19—N3—C18—C13	−65.20 (10)	C1—N1—C36—C31	58.97 (10)
C26—N3—C18—C13	170.97 (8)	C8—N1—C36—C31	−173.89 (8)
C16—C17—C18—N3	173.18 (8)	C34—C35—C36—N1	−174.29 (8)
C16—C17—C18—C13	−59.12 (10)	C34—C35—C36—C31	59.58 (10)
N4—C13—C18—N3	−50.19 (10)	N2—C31—C36—N1	45.54 (10)
C14—C13—C18—N3	−171.62 (8)	C32—C31—C36—N1	171.92 (8)
N4—C13—C18—C17	−179.93 (7)	N2—C31—C36—C35	173.06 (7)
C14—C13—C18—C17	58.63 (10)	C32—C31—C36—C35	−60.55 (10)

### Hydrogen-bond geometry (Å, °)

**Table:**

Cg1 and Cg2 are the centroids of the C2–C7 and C20–C25 benzene rings, respectively.

**Table:**

*D*—H···*A*	*D*—H	H···*A*	*D*···*A*	*D*—H···*A*
N1—H1···N2	0.90 (1)	2.32 (1)	2.7400 (11)	108.(1)
N1—H1···N4	0.90 (1)	2.12 (1)	2.8156 (11)	134.(1)
N2—H2···N3	0.88 (1)	2.19 (1)	2.9293 (11)	142.(1)
N4—H4···N3	0.88 (1)	2.33 (1)	2.7992 (11)	113.(1)
C1—H1a···O1	0.99	2.36	3.2096 (13)	143
C9—H9a···O3^i^	0.99	2.40	3.3620 (12)	165
C34—H34a···O3^i^	0.99	2.50	3.3876 (13)	150
C8—H8a···Cg3^ii^	0.99	2.53	3.4008 (11)	146
C26—H26b···Cg1^iii^	0.99	2.71	3.5899 (11)	149

Symmetry codes: (i) −*x*+1/2, *y*+1/2, −*z*+1/2; (ii) −*x*+1/2, *y*−1/2, −*z*+1/2; (iii) −*x*+3/2, *y*+1/2, −*z*+1/2.
